# Health literacy and cultural responsiveness of primary health care systems and services in Australia: reflections from service providers, stakeholders, and people from refugee backgrounds

**DOI:** 10.1186/s12889-023-17448-z

**Published:** 2023-12-21

**Authors:** Prince Peprah, Jane Lloyd, Mark Harris

**Affiliations:** 1 Social Policy Research Centre, University of New South Wales, Sydney, NSW 2052 Australia; 2 Centre for Primary Health Care and Equity, University of New South Wales, Sydney, NSW 2052 Australia; 3 Australia’s National Research Organisation for Women’s Safety, Sydney, NSW 2000 Australia

**Keywords:** Health literacy, Culture, Organisational responsiveness, Primary health care, People from refugee backgrounds, Access

## Abstract

**Background:**

Primary health care [PHC] services with general practitioners (GPs) as the first point of access to health care services for people from refugee backgrounds in Australia can play a crucial role in building health literacy and promoting access to culturally appropriate services. To achieve equitable access and engagement, services and systems must be responsive to diverse health literacy and cultural needs. This study aims to explore how primary health services respond as a system and organisation to the health literacy and cultural needs of people from refugee backgrounds in Australia.

**Methods:**

This exploratory qualitative study involved 52 semi-structured interviews among 19 Africans from refugee backgrounds, 14 service providers, including GPs and nurses, and 19 other stakeholders, such as service managers/directors. Participants resided in New South Wales, Victoria, and Queensland. Interviews were audio-recorded, transcribed, and coded into QSR NVivo 12. Data analysis was guided by reflexive thematic analysis.

**Findings:**

Three interrelated themes were identified from the data relating to the health literacy and cultural responsiveness of PHC systems and services. The first theme, *‘variable and ad hoc organisational response to health literacy and culturally responsive care,’* demonstrated that some organisations did not systematically address the inherent complexity of navigating the health system nor the capacity of services and providers to respond to the cultural needs of people from refugee backgrounds. The second theme, *‘individual provider responsibility,’* captured the individual providers’ interpersonal and relational efforts in supporting the health literacy and cultural needs of people from refugee backgrounds based on their motivation and adaptation. The third theme, *‘refugee patient responsibility,’* encapsulated people from refugee backgrounds’ adaptations to and learning of the health system to navigate and access services.

**Conclusion:**

Health literacy and culturally responsive practices need to be systematised by PHC organisations to be implemented and sustained over time. There is a need for diversity in the organisational leadership and health care workforce, organisational commitment, health literacy and culturally responsive care policies, provider training, and auditing practice as essential components of the change process. Engaging with refugee communities would allow services to focus on people from refugee backgrounds’ needs by design.

## Background

Globally, the number of people from refugee backgrounds, i.e., persons who live in another country as a result of fear of unreasonable persecution and are unable to return to their country of origin [1], has significantly increased in the last decade, with about 32.5 million people from refugee backgrounds living in the world (as of mid-2022) [2]. The change is mainly due to ongoing ethnic and political violence, civil wars, natural disasters, and political oppression. Over 70% of people from refugee backgrounds originate from five countries, including Syria, Ukraine, Afghanistan, South Sudan, and Myanmar [2]. Australia is one of the largest refugee resettling countries through its Refugee and Humanitarian Program. Between 1965 and 2023, Australia has resettled 947,636 people from refugee backgrounds (through both offshore and onshore programs), with the majority coming under the offshore component [3]. Among people from refugee backgrounds resettled since 2000, over 50,000 came from African nations, with the largest population coming from Sudan, Congo (DRC), Liberia, Sierra Leone, and Burundi. Most reside in regional and rural areas of New South Wales [NSW] and Victoria due to the Federal government’s regional resettlement policy [4].

People from refugee backgrounds have spent several years in camps and experienced higher levels of interrupted and fragmented schooling histories, discrimination, violence, harassment, and trauma throughout their migratory process [5, 6]. The extent and nature of their experiences in their home countries, migration journey, and Australia can affect them on biological, psychological, and social levels (bio-psycho-social). Their experiences also significantly impact their navigation and engagement with health services [7]. Upon their arrival in Australia, people from refugee backgrounds must navigate a complex health care system. This is difficult because of their limited English proficiency and unfamiliarity with the health care system and services. In addition, Africans from refugee backgrounds have reported experiencing institutional and structural racism, exclusion, and limited social integration and participation once living in Australia, further compounding the impact of their poor health status [5, 8].

The in-flow of people from refugee backgrounds have implications for primary health care [PHC] services and providers in most of the world’s host countries, including Australia, regarding capacity and capability [9, 10]. PHC systems and services remain entry-level to the mainstream health care systems in many parts of the world, including Australia. It is usually the first encounter that migrants, including people from refugee backgrounds, have with the broader health system in Australia [11]. Australia’s approach to people from refugee backgrounds PHC services is mainly mainstreamed and led by general practitioners [GPs] [12, 13]. Upon arrival in Australia, it is recommended that people from refugee backgrounds visit a GP for a comprehensive health assessment which involves physical examination, immunisation catch-up screenings and referrals, if necessary [14]. General practitioners also provide ongoing care to people from refugee backgrounds in private practices, hospital settings or community health settings in line with Australia’s primary care arrangement [15]. Australia also offers refugee-focused and gateway services for people from refugee backgrounds. State and territory-funded refugee-specific health services have been established to provide specialised PHC to people from refugee backgrounds in response to their complex health challenges and barriers to accessing mainstream health care. The services are also created due to difficulties experienced by many GPs in private practice in delivering care to refugees [16, 17]. Examples of refugee-focused health services include the New South Wales Refugees Health Service, NSW Service for the Treatment and Rehabilitation of Torture and Trauma Survivors [STARTTS], Refugee Health Program in Victoria, and Mater Refugee Health Services in Queensland [13, 18].

It is well documented that delivering culturally and linguistically sensitive and appropriate services is crucial in optimising health care access and improving equity in health outcomes for marginalised groups, such as people from refugee backgrounds [19]. Africans from refugee backgrounds are the most heterogeneous group with varied socioeconomic, cultural, and linguistic backgrounds [6, 20]. For instance, among Africans from refugee backgrounds, two individuals from the same religious and ethnic background can have significantly different languages, dialects, practices, perceptions, and understandings of health and information according to their value system, societal norms, geographical locations, and personal experiences [21]. Varied cultural histories, standards and practices, and language skills shape people from refugee backgrounds’ decisions and willingness to participate in social and economic life and access health services [22]. Cultural differences between providers and patients can contribute to contrasting views about health conditions and treatments, misconceptions, interpersonal conflicts, and poor health outcomes [23]. Also, previous research in Australia has shown that many patients from refugee backgrounds, especially Africans, have been excluded from health services, notably due to the cultural incompatibility of services [24, 25]. Thus, health systems and services must provide appropriate care to patient populations, such as people from refugee backgrounds with vastly diverse beliefs, values, and practices, considering Australia’s increasingly multicultural and diverse population [26].

Health literacy, defined as a broad range of skills and competencies to discern and act upon health recommendations and make meaningful decisions about health, is vital to good health and well-being [27, 28]. Health literacy is an essential element in PHC and a mediating determinant of the health and well-being of individuals and communities [29]. Health literacy is critical for patient empowerment, autonomy, and agency [30]. Health literacy has traditionally focused on an individual’s skills and competencies to access, understand, evaluate, and utilise health information to maintain health [31]. The health of individuals and communities is often influenced by the health systems and the critical competencies to navigate systems, analyse, and use health information meaningfully [31]. Unfortunately, limited health literacy disproportionally affects disadvantaged groups, such as people from refugee backgrounds in many parts of the world, including Australia [32]. People from refugee backgrounds with higher rates of interrupted schooling, limited English language proficiency, and limited social support and networks are at exceptionally elevated risk for poor health literacy [33]. People from refugee backgrounds, mainly Africans, face significant health literacy issues, such as difficulty navigating the Australian health care system and services, difficulty accessing medicines and pharmacy services, limited reading and comprehension of health information, and communication issues [20, 34]. Limited health literacy puts people from refugee backgrounds at higher risk of health issues, such as limited understanding of and participation in health care systems and preventive services, poor self-rated health, delayed diagnoses and treatment, chronic diseases and health service-seeking refrainment, and poor adherence to medical prescriptions [35].

Health literacy and culturally appropriate care are seen as collective practices of health systems and organisations working to accommodate needs, build knowledge and make the health system more user-friendly and accessible [31, 36]. Thus, one of the most critical responsibilities of health systems and services is to understand and address consumers’ health literacy and cultural issues, including those of refugee backgrounds. However, while health literacy and cultural issues, such as religious norms, language, social values, and ethnicity, are common among people from refugee backgrounds, especially those from African nations [21, 37], little is known about how health systems, including organisations and professionals, respond to the health literacy and cultural needs of patients globally. Meanwhile, various studies have reported that people from refugee backgrounds need organisational support to adequately navigate and access the complex health systems and services in the host countries [7, 38, 39]. For instance, one systematic review found limited evidence of strategies to address health literacy by health services [40]. More work is required to develop, test, and evaluate organisation-level interventions to support health systems to become more responsive to people from refugee backgrounds with low health literacy and diverse cultural needs. Suppose health systems and organisations are not designed to respond to health literacy and cultural diversity, those needing access and health literacy support will likely be left out or an afterthought. Therefore, this study explores how PHC systems and services respond to the health literacy and cultural needs of people from refugee backgrounds in Australia.

Organisational health literacy and cultural responsiveness are intertwined frameworks in the context of marginalised groups, such as people from refugee backgrounds [41]. For instance, the Institute of Medicine emphasises that a definition of health literacy without recognising the potential effect of cultural differences on health information communication and understanding will likely miss much of the more profound meaning and purpose of people’s health literacy [42]. McKee and Paasche-Orlow argue the connections between health literacy and culture, suggesting that health literacy should be understood through multicultural and multilingual lenses [43]. Health literacy and cultural responsiveness are systems rather than individual responsibilities [44, 45]. The health literacy and cultural responsiveness require systems and services to lower barriers to navigation access and respect and accommodate consumers’ distinctive sociocultural health beliefs and needs [46]. These ideas are relevant to this study because they involve Africans from refugee backgrounds with a higher prevalence of low health literacy and diverse cultural and linguistic needs. Health literacy and cultural responsiveness in Western health care systems and services like the Australian health care system and services are crucial due to the complexities of the systems, the dominance of Western biomedical models of care, and the vulnerability of specific health consumers, such as people from refugee backgrounds [46]. It is also essential due to the diversity of the population, emphasising the importance of services adapting and acknowledging the varying experiences, needs, and expectations of individuals and communities they serve.

Health literacy and cultural responsiveness may affect how Africans from refugee backgrounds from different health care systems and diverse cultural and linguistic backgrounds navigate, access/use, and engage with health care systems and services in Australia. This study investigates how PHC services respond to Africans from refugee backgrounds’ health literacy and cultural needs. The present study contributes to an evolving area of research by understanding how organisations and providers create an environment and services that practically responds to and supports Africans from refugee backgrounds’ health literacy and cultural needs. The study offers a better understanding of how services address the health literacy and cultural challenges of people from refugee backgrounds, which is lacking in many contexts. Better understanding would lead to improved access to appropriate services among people from refugee backgrounds. The study focuses on the PHC settings because it is the level of care that can adequately and flexibly respond to the complex and multiple health literacy and cultural needs of people from refugee backgrounds due to its unique characteristics and abilities, such as flexibility, comprehensiveness, accessibility, and social determinants of health-focused [47].

## Methods

### Approach

Data presented in this article is part of an exploratory-descriptive qualitative research project investigating the health literacy, cultural, and linguistic responsiveness of PHC organisations and providers to Africans from refugee backgrounds conducted by the first author [PP] between March and December 2022 in Australia. This design was used because how services respond to the health literacy and cultural challenges of people from refugee backgrounds in and outside Australia has received little attention [48]. A critical approach to the qualitative investigation was adopted, as it recognises the roles of inequality, injustice, and power in social, public, and population health research [49, 50]. Within this philosophy, the interpretivism paradigm guided this qualitative research [51]. Participants’ views about health literacy and culturally responsive care practices are relative and diverse and socially constructed intra-subjectively and inter-subjectively via experiences, meanings, and understandings accumulated within the lived social world, which leads to a kind of truth negotiated through dialogue [52]. This viewpoint is valuable for the population health perspective of health literacy because it promotes collective action to improve the public good in improving health literacy and culturally sensitive care, such as equity, social change, and justice [53].

### Study participants and recruitment procedure(s)

This study involved 52 participants, including professionals [14] from diverse PHC organisations across three states in Australia, including NSW, Queensland, and Victoria, other stakeholders [13], and Africans from refugee backgrounds [13]. Study-specific eligibility criteria for service providers included direct involvement in either management, program/policy development and service delivery concerning access and service navigation for at least five years. Stakeholders were included if they were involved in policy development, coordination, or program/service delivery concerning people from refugee backgrounds’ PHC access and service navigation for at least five years. Inclusion criteria for Africans from refugee backgrounds included living in New South Wales, being aged eighteen years or older, being able to communicate in English, and using PHC services in Australia.

Different non-probability sampling approaches were used to recruit the participants, including purposive, convenience, and snowballing. To recruit health professionals and other stakeholders, the study flyer was sent to health services and professional bodies working with people from refugee backgrounds. These services and organisations shared the flyers with health professionals who provide PHC care to people from refugee backgrounds. Those who received the invitations were followed up through emails, text messages, or phone calls to be given further details on the study and interview process and arrange a date for the interview if the participant was interested. People from refugee backgrounds were recruited via government and non-governmental agencies/organisations that either directly or indirectly provide services and support to migrants and humanitarian entrants. The organisations informed them about the study by sharing the flyer with them. Those who were interested were told to give their phone numbers to the contact persons in the organisations. With their consent, the phone numbers were forwarded to the first author [PP], who contacted them to brief them about the study further.

Initially, 77 people, including 20 providers, 32 stakeholders, and 25 Africans from refugee backgrounds, expressed interest in participating in the study; however, the first author could only contact 63. Reasons for non-contact included invalid contact details and no response to the first author’s calls, text messages, and emails. The final sample, 52, was because 11 people among those contacted dropped out. Due to the ethics requirements, those who refused to participate were not asked why they did not participate in the study. However, some willingly gave reasons such as unavailability and busy schedules.

### Ethics

This research was approved by the South Western Sydney Local Health District Human Research Ethics Committee (Ethics Approval Number: 2021/ETH11161). Informed consent was obtained from all subjects who participated in the study. Study participants were given an information sheet about the study. The participants could then ask questions before consenting in writing (face-to-face) before the interviews.

### Data collection

Fifty-two semi-structured interviews were conducted, each lasting 30 to 60 minutes. Semi-structured interviews were used to understand people’s experiences of health literacy and culturally responsive health service delivery practices and strategies. Due to the flexibility and reflexivity of semi-structured interviews, probes were used to explore emerging narratives during the interviews. Three semi-structured interview guides were designed for the health professionals, other stakeholders and people from refugee backgrounds, respectively, through a literature review [20, 37, 44], reflective supervision with supervisors, and following feedback from pilot interviews. These piloting and reflective supervision enhanced the study’s trustworthiness [54].

The provider and other stakeholder participants’ guides focused on what their services do to respond to the health literacy and cultural challenges of people from refugee backgrounds. The interview guide for people from refugee backgrounds focused on their experiences with the strategies services use to support their health literacy and cultural needs. All the interview guides collected basic background information such as the number of years providing care to people from refugee backgrounds, areas, country of origin, years spent in Australia, gender, and work location). Provider participants were then asked open-ended introductory questions such as: ‘What is your experience in providing PHC services to people from refugee backgrounds?’ Stakeholders were first asked: How are you involved in refugees’ service access and navigation? Both providers and stakeholders were also asked if they knew of any interventions or programs in their organisation supporting health literacy and culturally responsive care. People from refugee backgrounds were asked questions such as: Can you please tell me about your experiences visiting your health care provider? The preliminary questions allowed the interviewer to hear the participant’s experiences. Most interviews were conducted online (Zoom software). One interview was conducted in person.

The first author [PP] conducted all the interviews in English. Before the interviews, the first author had not met any of the study participants. He introduced himself as an international Ph.D. student in Australia who is originally an African from Ghana. He discussed his interest in PHC and migrant and refugee studies, including health literacy cultural and linguistic issues among Africans in Australia. Data collection was stopped after a realisation that thematic saturation had been met [55–57], and the thickness of the data gathered from the participants offered sufficient ‘information power’ to address the research aims and questions [58]. The interviews were both audio and video-recorded using the Zoom cloud recording function. Transcriptions of the recorded interviews were performed immediately after the discussions. The first author transcribed 36 out of the 52 interviews; the rest were professionally transcribed. The transcribers signed a confidentiality agreement. The transcripts were de-identified and checked with the recordings for accuracy.

### Data analysis

To effectively manage the transcribed data throughout the coding process, the transcripts were imported and coded into QSR NVivo 12, and thematic analysis was undertaken [59]. This method involves identifying recurring and differing patterns and themes in the data. As a result, detailed explanations of patterns of meaning were developed. In applying the thematic analysis approach, reflexive thematic analysis [RTA] following Braun and Clarke’s six steps was used as an iterative, inductive data analysis process [60]. This approach considers the researcher’s subjectivity, iterative data engagement, and introspection as an analytic resource in knowledge production. The coding process started with open coding, which generated a list of initial codes through inductive and deductive coding strategies. Then, through several intuitive and readings of the transcripts, additional necessary codes relating to health literacy and culturally responsive strategies and experiences were developed. After several reflective discussions and meetings, a comprehensive coding framework was developed for the analysis. The reflective discussions and meetings about the coding process and codes added to the study’s rigour [61]. Also, there was consensus on the essential codes to capture themes covered in the interviews. When there were differences in opinions among the research team about the codes, agreement was reached through further discussions. These codes were merged into potential categories, further collapsing into the main themes and subthemes. In presenting the results, participants’ voices were presented as quotes to add to the study’s credibility [62].

## Findings

### Participants’ demographic characteristics

The demographics of the study participants are presented in Tables 1, 2 and 3.

**Table 1:** Characteristics of service providers (*n* = 14)

**Gender**	
Male	4
Female	10
**Age (years)**	
30–39	5
40–49	6
50 and above	3
**Discipline**	
General Practitioner	4
Nurse Practitioner	2
Registered Nurse	5
Pharmacist	1
Psychologist	1
Paediatrician	1
**Duration of practice**	
10–15	4
16–20	3
20–25	4
Above 25	3
**State**	
New South Wales	8
Victoria	3
Queensland	3

**Table 2:** Characteristics of stakeholders (*n* = 19)

**Gender**	
Male	11
Female	8
**Age (years)**	
30–39	5
40–49	9
≥50	5
**Discipline**	
Service Manager	4
Service Director	3
Multicultural health worker	3
Resettlement worker	3
Liaison officers (primary health care facilitators)	4
Community elder	2
**Duration of role**	
10–15	4
16–20	6
20–25	4
>25	5
**State**	
New South Wales	10
Victoria	5
Queensland	4

**Table 3:** Characteristics of people from refugee backgrounds (*n* = 19)

**Gender**	
Male	9
Female	12
**Age (years)**	
30–39	5
40–49	10
≥50	6
**Number of years in Australia**	
≤5	4
6–10	8
11–15	5
> 15	4
**Stayed in a refugee camp**	
Yes	20
No	1
**Number of years spent in camp**	
≤5	6
16 − 10	10
11–15	3
> 15	2

### The identified themes

The analysis grouped the results into three broad themes, encapsulating the health literacy and culturally responsive care practices of PHC services: variable and ad hoc systemic response to health literacy and culturally responsive care (with two subthemes), individual provider responsibility (with two subthemes), and refugee patient responsibility. The themes are discussed below. In presenting illustrative quotes, participants were referred to PP1, RP1, and SP1 (provider participant 1, refugee participant 1, and stakeholder participant 1).

### Variable and ad hoc systemic response to health literacy and culturally responsive care

This theme relates to evidence that systemic responses to health literacy and cultural issues are variable and ad hoc. This theme has two additional subthemes: lack of systemic response to health literacy and cultural issues in mainstream services and specialised services’ ad hoc systemic response to health literacy and cultural needs.

#### Lack of systemic response to health literacy and cultural issues in mainstream services

There was no systemic response to people from refugee backgrounds health literacy and cultural challenges in mainstream services. Many providers and stakeholders stated that there needed to be a more systemic approach to addressing the health literacy and cultural needs of people from refugee backgrounds in mainstream services. All participants stressed that systemic interventions, such as providing training for professionals and implementing leadership-supported structures and multicultural policies could build, embed, and promote health literacy and culturally responsive practices across the core activities of the whole organisation. However, providers and stakeholders were unaware of any structural changes, interventions, programs, and strategies to respond to health literacy and the cultural needs of vulnerable patients like people from refugee backgrounds in mainstream services. All participants were explicitly concerned about this lack of or limited systemic interventions in mainstream systems and services.

*Honestly, it feels that there is no […] interventions by the organisations that specifically address the health literacy issues of patients, including refugees [….] the system is hard to change because if it changes, then things can change, you know. [PP 5]*

Some provider and stakeholder participants mentioned that there was a lack of systemic and organisational support, such as the lack of ongoing training for the health workforce in mainstream organisations:

*[*System changes should target] *all healthcare workers, but potentially the ones that belong to … the dominant cultural group in Australia, right? That perhaps providing more education, providing more opportunities for them to reflect and to explore their own cultural identity, their own cultural background. [SP 2]*

People from refugee backgrounds and stakeholder participants also highlighted the lack of diversity within health leadership and decision-making bodies. The interviews revealed that for service to be culturally responsive, more people from culturally and linguistically diverse backgrounds, such as people from refugee backgrounds, should be seen as clinical and organisational leaders to influence and make decisions that resonate with them. However, many stakeholder participants mentioned this was often not the case in mainstream services as decision-makers often do not share the experiences of marginalised consumers, such as people from refugee backgrounds.

*…I think one important thing is diversity in the workforce, most importantly* [among] *the decision-making people. Staff need to… reflect the communities and have members on decision-making committees so that they can influence policies and make sure that services are thinking what the communities are thinking… [SP 3].*

Many people from refugee backgrounds and stakeholders also lamented the absence of consultation and participation of marginalised communities, including Africans from refugee backgrounds communities, in planning, codesigning, delivering, improving, and evaluating programs and services that can lead to health literacy improvement and culturally responsive service delivery.

*I think also, it would be important for the service to try and find ways to build to establish links with peak organisations from, I suppose, the major cultural groups within that service… and to bring them on board, you know, to try and develop partnerships and, you know, really good positive working relationships. [RP 17]*

Provider and stakeholder participants stressed that a system response could promote the uptake of the above critical principles and practices of health literacy and cultural responsiveness at both individual and organisational levels. They explained that system change would facilitate unique approaches, such as teach-back, as they will become part of the organisational core health literacy and culturally responsive practices. However, since the systems were not changing, all participants consistently mentioned that these elements were often absent within their organisations.

*…so, system change is the first important thing to bring improvement in these people’s health literacy because* [once the system improves then] *the organisations can now help them. If the system is not changing, people cannot do enough… to help them because essential issues like policies and plans are not there. [SP 11]*

Due to the above narrative, providers from specialised services and stakeholders wanted mainstream systems and services to build their capacities to respond to people from refugee backgrounds instead of automatically referring refugee clients to refugee-focused services.

*when mainstream service providers here… have a refugee client coming in, they would they automatically want to refer to our service, but we would prefer that the system changes so that everyone feels capable of looking after… people with these kinds of diverse experiences… [PP 4]*

This subtheme demonstrates that mainstream services as a system lacked organisational level strategies and programmes to support the health literacy and cultural issues of marginalised populations such as people from refugee backgrounds.

#### Specialised services ad hoc systemic response to health literacy and cultural needs

This subtheme describes how specialised services as a system respond to people from refugee backgrounds, health literacy and cultural challenges, and the shortfalls in their organisational response. Specialised services resorted to ad hoc practices aimed at helping patients be more responsive and adapt to the system and services. Regarding health literacy, interviews with people from refugee backgrounds, providers from specialised services and stakeholders highlighted that some refugee-focused organisations scheduled home visits, hospital tours, and information sessions to support people from refugee backgrounds’ navigation and access to services. Information sessions mainly focused on topics such as the broader general knowledge of Australian health care systems and services, Medicare and health care cards, and appointments through different avenues, such as initial health assessments, home visits, and on-arrival orientations.

*…we visit them at their various homes when they arrive, and it is an excellent opportunity to inform them about our systems and services, such as Medicare and other health cards. We also do orientations about the health system and services such as primary and specialist health care, emergency, and ambulance… like how to book appointments and use interpreters… we do our best… so that people can get at least some information. [PP 10]*

The above quotes suggest that, although information sessions were an example of organisational responsiveness, they aimed to help people from refugee backgrounds cope with the system. People from refugee backgrounds and stakeholders stressed that information sessions might not be sustainable and effective in promoting health literacy of people from refugee backgrounds because they were often variable and had a selective reach. Some people from refugee backgrounds had not been informed about the Australian health care systems and services. Others were unaware of any information sessions being available since arrival.

*Absolutely, because it was a big cultural change for me, coming from Sierra Leone in a very small culture, close together. Coming to Australia, which is very big, like the cultural differences were huge and not know how to access the healthcare system, who to contact, and also maybe language barrier because sometimes… I think someone must be giving us information about how things work here, but we do not get that chance- I do not know why so there is a lack of information because, for instance, no one has educated us on how Medicare works. [RP 5]*

Apart from home visits, information sessions and orientations, people from refugee backgrounds, providers from specialised services, and stakeholders also cited efforts by refugee-focused PHC services to engage community stakeholders, including community leaders, multicultural health officers, community liaison officers, and religious leaders, to promote access to information and services. Providers from specialised services and stakeholders believed involving these stakeholders could yield fruitful outcomes due to the strength of trust between them and people from refugee backgrounds.

*…we try to engage key stakeholders like* [the] *community leaders and influential people within the communities… to help us share information with community members because I think they are the ones that the refugee community often trusts and go to when they have issues… [PP 4]*

However, it was revealed that the engagement and consultation were for sharing and increasing access to information among community members and not for creating programs and interventions that were likely to help services respond to the needs of people from refugee backgrounds, as commented below by some stakeholders:

*…what they do is that they just come to us to share information for them, but we do not sit down and plan with them to do things that will help community people to understand and maybe use services. We want to be involved but they don’t give us the chance to be part… [SP 7]*

Aside from relying on community stakeholders to promote information access, specialised services used community people as volunteers and bilingual community educators to educate and help people get into health appointments and access services.

*Our service also* [has] *like volunteers who are from the communities* [and they] *do* [a] *good job for us because they assist people to go to appointment… I think they are beneficial. [PP 11]*

Regarding culturally responsive care, providing cultural competence training, education, and orientation for health professionals to improve their cultural awareness skills and knowledge by health services were commonly mentioned among providers and stakeholders in refugee-specific services as strategies adopted to deliver culturally responsive services.

*…the service try…* [to] *organise cultural training and* [cultural] *competence orientation and education for staff, especially before starting work with the service… the orientation talks about using interpreters, communication issues and how… culture impact health. [PP 3]*

In most cases, such training and orientations were not refugee-specific and often offered in public health services and settings.

*There are some courses already online and that’s only the public system that doesn’t include any of the GPs or private specialists* [or] *pharmacists. And then within health, you don’t normally get the Ministry of Health saying everyone has to do this course. [SP 6]*

In addition to the above, stakeholder participants responsible for organising such training highlighted that most cultural training was non-mandatory and completed during onboarding as a one-off orientation—training in cultural issues for new staff needed to be mandatory.

[specialist service for refugees*] provides trauma informed training to providers but most of the trainings are not completed by the health care providers. This training must be compulsory, and [the] use of interpreters should be made compulsory. [SP 9]*

Collecting demographic information such as country of birth, spoken language, and religion of refugees was another strategy by specialised services to provide culturally responsive care.

[service’s operations include] *…documenting people’s country of birth, their language spoken, ensuring that we always offer a healthcare interpreter [when] English is not the first language of a person. We also have a separate program [with] multicultural health liaison officers who sit in the background and also do a lot of education for healthcare providers. [PP 1]*

Some stakeholders with refugee backgrounds emphasised the complexity of refugee patients’ backgrounds and the need to carefully collect information on a full range of characteristics such as language, country of birth, migration history, and need for an interpreter:

[There is an assumption that if*] … you are black, you might be from Africa […] They don’t know my place of birth. What language I speak […] Huge recommendation from me is collect data, collect demographics about country of birth, language spoken at home and whether the person needs [an] interpreter and possibly migration history. So you know, if the person is here as a migrant who come to marry someone, his issues are so different from a refugee who came from who tortured and came from the war torn country. [SP 9]*

Theme one demonstrated that while there were some organisational responsibilities in specialised services though ad hoc, there was a lack of systemic response to health literacy and cultural challenges of people from refugee backgrounds in mainstream services.

### Individual provider responsibility

The second overarching theme reflected clinicians’ working in PHC interpersonal and relational efforts and strategies in supporting people from refugee backgrounds’ health literacy and cultural needs based on their motivation and adaptation. The theme also described the complexities and gaps in these individual providers’ strategies. The strategies included communication and health education, described as subthemes below.

#### Communication strategies/practices

Providers from specialised and mainstream organisations highlighted some communication strategies in helping people from refugee backgrounds overcome some of the health literacy and cultural challenges they experienced in accessing mainstream services.

*…there is nothing like system responding to refugee people’s health literacy because it is about you, the nurse, who has to be aware of the issues and your responsibilities… you have to use teach-back and language services* [and] *translated information more often… one problem is that not everyone can do this, for instance, those outside the refugee clinics and hospitals* [because] *they do not have the time. So… it is not about the systems or organisations and their duties to engage and support people who are vulnerable like refugees, and* [so] *we ran into problems with communication in how appointments* [are] *communicated to clients. [PP 4].*

Providers from both mainstream and specialised services talked about using different communication methods when communicating with people from refugee backgrounds to check and test for understanding of information. To address health literacy, providers used a technique involving recall or teach- back (asking patients to repeat back to the provider what they have understood from the conversation as a test of how well the provider has communicated to the patient).

*I use teach-back often because you can’t be sure… that people understand what you want them to do so you need to make sure you do some repetitions of important points to identify what they need and support them appropriately. It is a good way of checking…* [that] *people understand what you have told them or not, but I think not everyone will have the time to do this because we are limited by time… [PP 10]*

Many providers, particularly those in specialised services, specifically commented on the use of an interpreter in terms of attempting to address language barriers:

*… when we* [are] *seeing people*, [we use] *interpreters.* [We] *try and have time, so* [that] *we can listen to people and not* [to] *rush into things. [PP 10]*

The above comment implies that using communication practices like teach-back and interpreters ensures interpersonal communication and interaction, including how health information was communicated verbally and non-verbally between providers and patients about health and health care, not about addressing system issues. The provider participants further highlighted the complexities of these communication practices. The below quote further emphasises this point:

*Things become very complex in having to organise travel and interpreters’ overnight… and these are… people who don’t have the capacity to organise those things themselves. It is never simple, and everybody is special and different and has their own commitments, so trying to help them navigate for the best outcome is sometimes very time consuming. And of course, the constant need of interpreters. Just because someone nods and smiles nicely doesn’t mean to say they understand yeah and trying to provide health literacy or health information. It is a challenge, because, particularly with refugees, we often get people with new and emerging languages, and so they don’t actually have access to interpreters. So it gets very complex, very complex. [PP 4]*

Many refugee and stakeholder participants revealed that many providers in mainstream services do not have the skills and experience to use teach-back. One stakeholder explained perceived inappropriate understanding and use of teach-back in mainstream services:

*That’s a very good point again. What can I say about the theory of teach back? It’s all about testing yourself, not the client. It’s how people see it because often, especially not so much primary healthcare level like nurses… but once you get to doctors and specialists… it’s all about I have knowledge to give* [it] *to you. It’s a one way stream, and I think* [that is] *what is happening much more. You can’t change, it* [is] *like that there’s a push to say no engagement. [SP 17]*

Providers’ communication actions reflected a limited view of health literacy and cultural responsiveness ─ the leadership within PHC services, particularly mainstream services, assumed that interpersonal communication by health professionals was sufficient to improve health literacy and culturally responsive care, as reflected in the comment below:

*It is about making people understand enough. So basically, we cannot do enough than to help those who do not have the capacity like the refugees to navigate a very bad and complex system… so* [a]*part from teach back and others there are no interventions as far as I know.*

The above quote further indicates providers’ frustrations and complexities in supporting people from refugee backgrounds’ communication needs. It highlights the impact of the lack or ad hoc systemic response to people from refugee backgrounds’ health literacy and cultural issues on health professionals’ service delivery. In the quote below, a provider from a specialised service describes a specific example of the difficulty arranging a referral for a patient:

[I am] *in* [a] *major meltdown with telehealth, client, psychiatrist*, [and] *interpreter from TIS* [The Interpreter Service]… *sometimes just nothing goes right. Just trying to defer an appointment today with the neurological team in John Hunter Hospital – how the hell do they think the average person can do it, let alone someone with limited English? No wonder so many appointments fall over and are not economically viable. [PP 4]*

The narratives indicated that though health professionals were trying to support the information and communication needs of people from refugee backgrounds, their efforts have some limitations/gaps.

#### Health education

Health care professionals described their technique of educating patients from refugee backgrounds about their health, treatment options, and the potential consequences of each treatment option during consultation. The visual demonstration approach appeared valuable for providing clinical information to people from refugee backgrounds.

*…I find drawing pictures really helps because they’re* [from] *a farming community*…[and they have]*… a pretty good idea of anatomy. So, I can draw a picture of the human body… and explain things, and that works quite well… So, images and audio are good… [PP 5].*

The quote below suggests that time is a limitation that might not allow for patient education in mainstream PHC services. This result implies that time may influence routine activities, such as health education of mainstream organisations. In this context, health education is based on the motivation of individual providers, as commented below:

*…they were always in a rush… a lot of people waiting. So, I don’t think that they had the time to sit… and talk with you. If you sit me down and you teach me ABC D, I will understand. I know it might take time, but it is better that way. But what… is the problem is that* [the] *doctors* [and] *clinics do not do that always. Sometimes, you will meet a doctor who will get time to explain things well for you to understand, and other times, you* [will] *go… and meet a different person, even in the same clinic. I think it is because education is not part of what they do or is not compulsory for them to do. [RP 5]*

The above comment suggests that patient health education may not be sustainable and effective in empowering patients due to its unplanned, irregular, and ad-hoc nature. Thus, while some people from refugee backgrounds may have education about their health, treatment, and care process, others may not have the opportunity. Therefore, providers’ efforts were limited by their inability to cause a sustainable change in health literacy and culturally responsive care.

### Individual refugee patient responsibility

This theme presented the implications of the variable and ad hoc systemic response and individual provider responsibilities based on their motivation for patients from refugee backgrounds’ health system and service navigation and access. Participants from refugee backgrounds described an implicit assumption within the PHC health care system that makes the responsibility to navigate systems and services fall to them. This finding contrasts with the concept of health literacy responsiveness, where health systems are seen as responsible for responding to the health literacy of people from refugee backgrounds by making changes to address its problems, including reducing health literacy burdens and demands on refugee patients to facilitate better navigation, access, and engagement. One participant from a refugee background expressed frustration with mainstream health services and perceived lack of care, attention, and time to supporting patients’ health literacy and system navigation:

*The fact is that no one cares about poor health literacy… no one cares my brother. No one cares if you understand Medicare or can navigate services. So, it is all about you trying to learn what you can learn to be able to understand a few things and navigate and use services when you need. You need to go through the hardships and for me it is not the system helping people to understand. If you can’t speak English over here, then it is very worse for you. No one has time for you. [RP 16]*

Many participants from refugee backgrounds felt that mainstream PHC health systems and services assumed that all patients had adequate health literacy. Thus, they assumed that patients from refugee backgrounds could take responsibility for their self-management and navigation.

*…I think they assume everything. They should see that we are not from here and get time to help us. Let us share the responsibilities, and that will help. If you’re raised here, then you know it’s a very simple system… [RP 16]*

Another participant from a refugee background focused on a misunderstanding of patients’ health literacy abilities by mainstream PHC services and providers. In the quote below, the patient feels misunderstood by providers and the service and frustrated that an intervention, such as translating materials online, is seen as sufficient for improving a complex concept, such as health literacy and cultural responsiveness:

*It is difficult, it is difficult… What makes me sad is they think we know, they think we can access the system like them, they think it is our responsibility to look for information* [and] *understand the information. So, they will keep on saying we have translated information online*, [but] *how do you go to the online when you are not capable? They should know it is their problem and they need to help us. [RP 17]*

Some participants from refugee backgrounds further shared their frustrations about the assumptions in mainstream PHC health services’ policies or procedures and among professionals regarding their abilities to access services and information.

*What is very serious is that the doctors and hospitals think that people can find information on their own, they can understand, and they can use the information, but the truth is* [that] *most* [of] *us do not know how, where, and when to find the information. [RP 1]*

Participants from refugee backgrounds felt that the efforts by mainstream PHC services and providers were insufficient, and universally responsive health services did not exist. This failure results in the patients having the ultimate responsibility to identify ways to access and navigate health care. This sense of individual patient responsibility was experienced on several levels. For instance, asking for professional interpreters was often assumed to be the patients’ responsibility, rather than the service/organisational taking responsibility for identifying their language needs and providing support.

*…a lot of them didn’t know about things like… interpreters, and some are afraid of asking for interpreters*, [but] *if you do not ask, you will not be given. [SP 12]*

All the participants from refugee backgrounds lamented the mainstream PHC systems’ resistance to change to respond to their health literacy and cultural needs.

*Something must be done because it is difficult… as a nurse, there is a general thing that nothing changes easily in terms of the system. To me, it is a big problem for people like refugees to… use services and for services to respond or support them. [RP 5]*

Some stakeholders also felt that, in many cases, the mainstream PHC systems and services expected people from refugee backgrounds to adjust rather than take responsibility for changing and responding to the health literacy issues of vulnerable groups.

*…what… often frustrates me is* [that] *the health system sits here and thinks everyone has to slot* [in] *rather than understanding that they need to change, they need to respond adequately to bring change… but… it seems that it cannot change to make service respond to people which is the problem. So, you know there is a deficit… in the system… [SP 13]*.

Participants from refugee backgrounds spoke about the difficulties they experienced in taking responsibility for their navigation, access, and engagement.

*…another* [thing] *is that the hospitals* [that] *we go… think we know everything because I think they…* [do] *not know that we are not from here. Sometimes you can miss your appointment because you do not know which place to go… when you tell the doctor that you struggle to come here, they* [will] *say I know, yes I know it* [is] *like that always. It is challenging… [RP 11]*

Some participants from refugee backgrounds, especially non-English-speaking backgrounds and with limited education, further shared their experiences of the mainstream PHC system’s resistance to change and the perceived lack of systemic response to health literacy and cultural needs on their health care navigation, access, and utilisation. Some talked about the difficulty in navigating the online systems for booking appointments and finding information.

*Everything is* [on] *google and online, but how can someone* [like] *the old ones who have spent decades in camps look for information online in English* [?] *how can he even book an appointment online, even how can he or she access the online in the first place*[?] *[RP 17]*

Others also decided to avoid seeking the mainstream services they needed in the long run because they lacked system responsiveness. One stakeholder participant from a refugee background mentioned that:

*There are others who decide not to go either* [because] *they cannot book an appointment or cannot express themselves well due to language* [issues] [SP 10].

Some provider participants had observed refugee patients missing out on care because of difficulty navigating care:

*People don’t go and get that X-Ray or the blood test or see the specialist or get those medications because they don’t understand how to navigate to do all… those things. [PP 10]*

Another stakeholder participant also added to the above viewpoint:

*…that’s why you see so many refugees do not use the services and are struggling with so many health conditions because they cannot navigate the health system.* [SP 10]

This theme highlighted the responsibilities of people from refugee backgrounds in terms of service navigation and access. It further demonstrated that people from refugee backgrounds faced challenges in navigating and accessing services and information due to the lack of systemic response to health literacy and cultural needs within mainstream PHC systems and services. All the quotes in this theme are referring to health services within PHC though some of the quotes suggest services beyond PHC setting.

## Discussion

This study provides insights into how PHC organisations and providers respond to people from refugee backgrounds’ health literacy and cultural needs. We identified three interconnected themes, including variable and ad hoc systemic responses to health literacy and cultural needs leading to individual provider responsibilities of communicating information appropriately and educating patients based on their motivation and individual patient responsibilities regarding navigation and access. Patients and providers in this study felt there was a lack of systematic responses to the health literacy and cultural needs of people from refugee backgrounds in mainstream PHC systems and services in Australia. To navigate and access services, people from refugee backgrounds had to learn and adapt to the health system and services, which often resulted in issues such as missing out on care. This finding is particularly concerning considering the vulnerability of people from refugee backgrounds, particularly those from African countries [37]. People from refugee backgrounds are part of the minority group with different cultural practices and languages in Australia. Most Africans from refugee backgrounds from culturally diverse backgrounds believe in other modalities of care [21] and have experienced health systems where they do not need to book an appointment online before seeing providers or seeing a GP for a referral before attending a medical specialist. They also communicated in their native language with health professionals [63]. This experience contrasts Australia’s multiple and layered health care systems and services that essentially promote economic efficiency. The reported access issues among people from refugee backgrounds in Australia [25] could be partly explained by the limited or lack of system-level responses to health literacy and cultural needs in mainstream services found in this study. Making changes in the mainstream health system and services, including greater policy attention on health literacy and cultural responsiveness, could help promote service responsiveness for all, mainly patients with low health literacy and those from culturally and linguistically diverse backgrounds, such as Africans from refugee backgrounds [64].

Examples of a successful systemic response to health literacy can be found in Melbourne West [65] and regional health services in New South Wales [66, 67]. Health literacy was strategically identified as a priority, and health literacy development projects with training courses incorporated targeted services within health and community systems. For instance, the Illawarra Shoalhaven Local Health District in New South Wales developed an evidence-based health literacy framework and embedded health literacy into its regional health systems by creating a supportive web-based platform and governance structure for designing and validating plain language. The district also outlined a process for consumer engagement, developed health literacy ambassador training programs, and integrated health literacy clinical quality improvement procedures through a well-recognised program with health consumers to direct processes, including hospital navigation and improvement in access to health care services [67]. Health literacy was seen as both a process and an outcome, and the coordinated approach adopted by this LHD to respond to health literacy has been recognised by policy makers and institutions, such as the Australian Commission on Safety and Quality, as an exemplar of a coordinated way to embed health literacy into health systems [67].

There were ad hoc organisational strategies and practices to support people from refugee backgrounds. However, these strategies and practices were more seen in specialised services. Regarding health literacy, services adopted some techniques and practices, such as outreach visits, information sessions about the Australian health care system and services, orientations, and community connections, to help marginalised groups, such as people from refugee backgrounds, understand and cope with the system better. There was also service or organisational response in specialised services regarding culturally responsive care, such as the uptake of generally non-mandatory cultural competence training and using interpreters and bilingual and cultural liaison health workers. These practices have been termed ‘Band-aid solutions’ because they are temporary and may not be enough to address the health literacy issues of people from refugee backgrounds structurally and sustainably [68]. Relying on the ad hoc responsibility being taken by services does not address the inherent complexity of navigating the health system nor the capacity of providers to respond to the health literacy and cultural issues of people from refugee backgrounds. Applying these solutions to help patients adapt to the system and access services is unlikely to be sustainable, reach all people from refugee backgrounds, and be effective without complementary system changes. For instance, implementing these strategies is random, selective, and location-specific as some people from refugee backgrounds have yet to be visited by refugee health services or have an orientation about the Australian care systems and services (a critical component of the Australian Humanitarian Settlement Program [HSP]).

We also found that providers, particularly those in specialised services based on their motivations and goodwill, needed to adapt communication strategies such as using interpreters, teach-back, and health education to support patients’ communication and information needs. Generally, the literature supports using methods such as interpreter use, patient health education, and teach-back technique to support health literacy and cultural needs, especially among marginalised groups like people from refugee backgrounds [69]. For instance, in the context of refugees, some studies have reported that access to information about the health system and services remains an essential factor in their health and well-being. Others have also reported that education by health services and professionals is critical to people from refugee backgrounds’ health literacy [70]. Various studies have found the teach-back technique to be an evidence-based communication method that promotes two-way participation in the care process, improved medication education and comprehension satisfaction, self-care abilities, discharge information, and health management [71]. Previous refugee studies have also reported some perceived benefits of cultural competency training, such as helping professionals consider people’s culture, respect people’s cultural practices, and acknowledge the impact of unconscious bias through self-reflection [72]. Thus, providing navigation support, using interpreters, and educating patients about the health system may help people from refugee backgrounds agency to incrementally build change within their sphere of influence and control in their day-to-day activities within the health context, such as accessing and navigating services.

Nevertheless, more than these solutions are needed. For instance, in line with previous evidence, our results suggest that some major problems with these practices are that health organisations and providers cannot deliver them, their adoption and implementation are highly variable, and insufficient resources to sustain them [64, 73]. We argue that unless policy-makers and leadership within health services, particularly mainstream PHC systems and services, make comprehensive system and policy-level changes by making health literacy and culturally responsive care a priority and developing targeted projects, their efforts to translate materials, use interprets, provide cultural training, and teach back though necessary might not be enough to address poor health literacy and cultural issues of marginalised populations, such as people from refugee backgrounds. These approaches can be a good start on health literacy and culturally responsive care improvement. Health systems and organisations can implement these strategies to create a systemic response and change. For example, health literacy is not fixed, and progress can be incremental; it can be developed over time through appropriate environments and interventions by health organisations [74]. Health systems and organisations can build on simple health literacy practices to reduce the demand on patients from refugee backgrounds’ skill levels required to navigate services. For example, simplifying the process involved in attending a specialist service (e.g., fewer forms to complete) and having bilingual community health navigators in general practice may be desirable to facilitate navigation, access to services, and build capacity [75]. These strategies would systemically create change to support the health literacy strengths and limitations of people from refugee backgrounds over time [69, 76].

Similarly, ad-hoc strategies, such as cultural competence training and bilingual and cultural liaison health workers, can be a starting point or basis for developing and implementing more significant systemic and organisational responses to people from refugee backgrounds’ cultural needs and expectations. Integrating bilingual and cultural support workers into the mainstream health workforce and addressing the non-mandatory nature of cultural training, orientation, education, and interpreter use would be a reasonable basis for embedding cultural responsiveness into health organisations [77]. A previous study found that health professionals perceived cultural training as optional and of less importance [78]. Mandating ongoing cultural training and orientation would help raise its priority.

The contents of the presented themes are firmly interconnected, and they have a dynamic relationship. For instance, an ad hoc systemic response includes how providers can fulfill their responsibilities, influencing refugee patient responsibilities. Individual provider responsibilities are determined in part by the provider’s goodwill and the health care system’s complexity [69]. The more complex a system is to navigate, the greater the good will of the provider is called upon to support patients from refugee backgrounds. The providers’ efforts are also frustrated by the system’s complexity because if the health system is not fit to help patients with lower health literacy and from diverse cultural and linguistic backgrounds, then there is a greater reliance on the good will of providers. Also, the burden falls on patients if systems and providers cannot provide health literate and culturally responsive health care [74]. This results in missed appointments, failure to understand critical information and a lower standard of care [39]. Thus, there is a dynamic relationship between system, service, and patient responsibilities. Failures in one area of the system will likely have ripple effects on providers and patients [7, 76].

### Conceptual and practice implications

The study findings have important implications for health literacy and culturally responsive care. On the conceptual level, a lack of system-level response to health literacy will likely improve functional rather than critical levels of health literacy, where patients develop agency, autonomy, and empowerment [30]. The health literacy responsive practices identified in this study do not meet the theoretical threshold of health literacy responsiveness requiring organisations to establish infrastructure, structures, processes, and interventions that minimise navigation and access barriers, enable communication, reduce the level of health literacy needed to access and navigate their services by people with low health literacy, and improve the use of health care for all patients, irrespective of gender, race, culture, and other sociocultural elements [74]. Again, in a policy context, this study’s health literacy responsive strategies do not match those envisaged in policy frameworks. For instance, the New South Wales health literacy Policy 2019–2024 posits that it is the responsibility of organisations to simplify information, check in to ensure effective communication and understanding, make health systems and environments easier for every patient to navigate, and support patients to self-manage their health and well-being to improve health literacy [79].

However, building health literacy requires both incremental and systemic change within the health systems and organisations. Incremental change should target marginalised groups, such as people from refugee backgrounds; efforts to improve the health literacy of patients through information sharing, education, and effective communication are essential to increase agency over day-to-day health activities within their control [80]. Systemically, engaging governments and prioritising health literacy in plans and communication about health issues and codesigning solutions, programs, and interventions with marginalised health consumers, including people from refugee backgrounds that advance health literacy by health services, are essential [81]. This idea is supported by health literacy responsiveness studies that recognise that a whole-of-society approach to health literacy is needed to remedy health literacy issues [82].

Just as health literacy, for culturally responsive care to occur within practices, services must consider a whole-organisation approach in implementing existing techniques such as cultural competence training and orientation. Inclusive leadership is required at the highest level of services. The clinical and non-clinical staff should be given the opportunity and resources for professional development to enhance their cultural skills and capabilities. Also, PHC delivery systems should be based on inclusive care design and planning practices, especially within mainstream services. Finally, codesign, evaluation, and review of culturally responsive service strategies and interventions within health organisations are essential to delivering culturally responsive services to people from refugee backgrounds. The above considerations should not be implemented in silos but supported by high-level strategic planning and governance, reporting monitoring, and evaluation procedures and processes [78]. It must be emphasised that implementing the above suggestions by services cannot realistically address all cultural issues and needs of people from refugee backgrounds. However, they can help towards a better and more informed understanding of culture and its essential role in people from refugee backgrounds’ care and outcomes.

### Strengths and limitations

Strengths of the study included the inclusion of multiple perspectives indicating the diversity and variation in the accounts, adding to the study’s credibility, rigour, and comprehensiveness of findings [83]. While the findings of this study offer valuable insights into health literacy and culturally responsive care practices of professionals and organisations at the PHC level, the study has some limitations. All the participants from refugee backgrounds were recruited from Sydney, and none came from Australia’s regional and rural areas. Also, the participants from refugee backgrounds were all Africans who could communicate in English though they came from nine different African nations. Thus, the perspectives of people from refugee backgrounds are restricted to the accounts of Africans who are fluent in English residing in Sydney. We acknowledge that people from refugee backgrounds with limited or no English proficiency may have different experience. Also, only a few provider and stakeholder participants came from PHC services in regional and rural areas. Thus, the findings may not represent all PHC services and need to be viewed and interpreted in this context. Also, the study recruited and interviewed providers from specialised services who were actively engaged in promoting people from refugee backgrounds health literacy and service access. People from refugee backgrounds who go to services without specific positions and resources to support refugee/multicultural patients would probably have much worse experiences. Similarly, service providers and stakeholders at those services would have different views about what they do to support people from refugee backgrounds. Also, we did not perform member checking of the study data or findings.

## Conclusion

Responding to the health literacy and cultural needs and expectations of culturally and linguistically diverse groups, such as Africans from refugee backgrounds, is complex and challenging for health services and professionals, particularly mainstream services, and providers. This study has provided evidence on health literacy and cultural responsiveness of PHC services to people from refugee backgrounds in Australia. The findings suggest that more work is required for health systems to respond to people from refugee backgrounds’ health literacy and cultural needs. The finding is a clear example of a health care system not designing and providing health literacy and culturally responsive care systems and services but instead providing temporary solutions to issues that need a systemic and structural response. The findings call for a reflection on how systems are organised, processes and structures are laid down, and services are delivered within health services for people from refugee backgrounds. This reflection is not only a task for health professionals but ultimately a concern and responsibility across all levels of health systems and organisations [38]. Also, a fundamental shift is needed in the design and delivery of health organisations if services are to be responsive. This shift may include making health literacy and cultural issues strategic priorities, having culturally inclusive service leadership and governance structure, employing a diverse workforce that reflects the population they serve, training the workforce, and setting up outreach services for people from refugee backgrounds. Also, compulsory implementation of existing health literacy and culturally responsive strategies can provide the basis and avenues for health organisations to respond to the health literacy and cultural needs of people from refugee backgrounds in the short term and create a more systemic and organisational response in the long run. Doing so can promote access to health care and improve health outcomes through patients’ empowerment, activation, and agency.

## Data Availability

All datasets used and/or analysed during the current study are available upon request. Please contact the corresponding author [Prince Peprah] if you want to request the data from this study.

## Abbreviations

GP: General Practitioner(s)
NSW: New South Wales
OHLR: Organisational Health Literacy Responsiveness
RTA: Reflexive Thematic Analysis
